# **Exploring the Psychosocial Experiences of Individuals with Developmental Language Disorder During Childhood: A Qualitative Investigation**

**DOI:** 10.1007/s10803-023-05946-3

**Published:** 2023-06-20

**Authors:** Annabel Burnley, Michelle St Clair, Charlotte Dack, Hannah Thompson, Yvonne Wren

**Affiliations:** 1 Department of Psychology, University of Bath, 10 West, Claverton Down, Bath, BA2 7AY UK; 2 Bristol Dental School, University of Bristol, Bristol, UK

**Keywords:** Developmental language disorder, Specific language impairment, Parents, Children, Psychosocial difficulties

## Abstract

Children with Developmental Language Disorder (DLD) often experience co-occurring psychosocial difficulties, the developmental trajectories of which are still not fully understood. This study sought to explore the manifestation of such difficulties during childhood, through first-hand accounts of those with DLD and their close relatives. Individual semi-structured interviews were conducted with 11 mothers of children with DLD (aged 6-12 years old) and were analysed alongside the secondary data from interviews of five adults with DLD. Interviews were conducted online; all participants resided in Europe and were fluent in spoken and written English. A process of interpretive phenomenological analysis resulted in the development of five overall themes: experiencing anxiety, social frustrations, maintaining factors, childhood strengths and the parenting experience. Cognitive appraisals appeared particularly important during childhood in both escalating and maintaining anxiety, low self-esteem, emotion dysregulation and social frustrations. High levels of isolation and stress were experienced by all mothers. The findings suggest parents in the United Kingdom and Ireland require more support and guidance at the point of diagnosis than is currently provided. Emphasis was given to the link between children’s experience of anxiety and social behaviours, such as withdrawal, as well as their intolerance of uncertainty. Internalising symptoms were a prioritisation for intervention during childhood by both parents and adults with DLD.

Developmental Language Disorder (DLD) is a common neurodevelopmental disorder, estimated to impact 7.6% of children (Norbury et al., 2016). It is characterised by expressive and/or receptive language difficulties, however these children are also recognised to commonly exhibit co-occurring psychosocial difficulties (e.g. Conti-Ramsden et al., 2006). Literature has suggested that those with DLD can experience six times the rates of anxiety (Yew & O’Kearney, 2013), twice the rate of hyperactivity (Mueller & Tomblin, 2012), are more socially withdrawn (Hart et al., 2004) and struggle more often with peer victimisation than their neurotypical peers (Gerbig et al., 2018). This study aims to understand the relationship between the different psychosocial characteristics of children with DLD, and their manifestation; a crucial step in improving the management of symptoms.

Suggestions have been made that psychosocial difficulties develop in individuals with DLD in a similar way to those with autism spectrum disorder (ASD; Francis et al., 2022). For example, the social information processing theory indicates that the presence of ASD or DLD impacts the social cognitive abilities required for successful interactions with neurotypical peers (Crick & Dodge, 1994; Russo-Ponsaran et al., 2015); leading to more social withdrawal (Rubin et al., 2010) and social anxiety (Yi et al., 2022). The wealth of research investigating the manifestation of psychosocial difficulties in children with ASD (e.g. Magnuson & Constantino, 2011; Mayes et al., 2021; Ozsivadjian et al., 2012; Rowley et al., 2012) has led to the development of many tailored psychosocial interventions (e.g. Lerner et al., 2012; Seida et al., 2009). Thus far, there has been relatively limited investigation into the relationship between these co-occurring difficulties for children with DLD; most likely due to the lack of attention DLD receives in research (McGregor, 2020). For example, DLD is often neglected in psychopathological reviews of children with neurodevelopmental disorders (e,g. Baraskewich & McMorris, 2019). It is important to understand in more depth how the development of psychosocial difficulties amongst children with DLD compares to other neurodevelopmental disorders.

The social anxiety experienced by children with DLD has been tentatively linked with their difficulties in navigating or interpreting social situations (Wadman et al., 2011). However, comparative studies dispute this claim, suggesting more of an association between low IQ and social anxiety (Snowling et al., 2006). Building on this, hypotheses have also suggested connections between the negative social experiences of DLD (e.g. through victimisation) and the development of negative cognitive appraisals (i.e. negative perception of another child’s social response; Timler, 2008), which are symptomatic of internalising symptoms (e.g. Alvares et al., 2012). If true, relevant therapies could be tailored to help those with DLD manage misplaced negative appraisals (e.g. cognitive behavioural therapy; Fenn & Byrne, 2013), however without consensus, the success of such adaptation is limited.

More recent literature has begun emphasising underlying psychosocial differences that may contribute to the development of these children’s many problems. For example, van den Bedem’s work has suggested that it is the children’s difficulties in regulating their emotions that could either lead them to adopt more avoidance strategies (e.g. social withdrawal; Bedem, 2018), or lash out aggressively in-the-moment (Bedem, 2020). Further research has supported this latter suggestion, that aggressive behaviours are more likely due to poor conflict resolution, rather than calculated rule-breaking (Winstanley et al., 2018). Nonetheless, this research remains speculative.

One of the potential issues with the current research landscape is the lack of in-depth qualitative work with DLD populations. The strict and structured nature of quantitative research may limit novel insights into the development of these psychosocial issues, and how best to intervene. Furthermore, the marginalisation of individuals with DLD is only worsened when their own personal experience is negated (Gillies, 2000; Lloyd et al., 2006). Adults with lived experience of childhood with DLD can offer insight that the children themselves may struggle to reflect on or express (Perry & Felce, 2002). These include (I) the parents of children with DLD, and (II) the memories of adults with DLD.

Parents are the relative experts of their own children (Glascoe et al., 1991). Moreover, the family context is acknowledged to render a large influence over the psychosocial development of such children from a young age (Assous et al., 2018). Qualitatively working with parents therefore provides an opportunity to build a further understanding of their child’s difficulties, and discuss their unique social and developmental contexts; additionally important given the recognised heterogeneity of the DLD population (Bishop, 2017). Patient-centred approaches to research such as this are becoming increasingly valued, and even required for intervention development (Evans, 2006), marrying the evidence-base with lived experience (Gillard et al., 2012).

This study has two main aims: firstly, to qualitatively investigate parents’ perspectives on the psychosocial difficulties of their children with DLD (study I); secondly, to qualitatively understand the psychosocial experiences of children with DLD, from the perspectives of adults with DLD (study II). In doing so, the study will provide more patient-centred evidence for the development of an appropriate intervention by representing the needs and priorities of individuals with DLD who are currently underserved by public health services (e.g. Rannard et al., 2005).

## Methods

### Participants

All participants were given pseudonyms, and all other potentially identifying details were removed (e.g. occupations).

Eleven mothers were recruited for study I (aged 27–43 years old; Mean = 38.5), all of whom were female, and lived in the UK (n = 9) and Ireland (n = 2). Initially, convenience sampling was used, followed by purposive sampling to ensure that a wider range of life contexts were reflected. These mothers described their ethnicities as White British (n = 6), White European (n = 4; including Irish, Lithuanian, Turkish and German; two were bilingual) and Black British (n = 1). Two of the mothers were single parents, with all others cohabiting with their partners. For those who disclosed their household income, two families had below average income (below £30,000 p/a), three had above average income (£30–60,000 p/a) and two had incomes in the top quintile of UK income (more than £60,000 p/a); four mothers declined to disclose their incomes.

Those residing in the UK covered a range of counties across England and Scotland, including Cornwall, Greater London, the Isle of Wight, Lanarkshire, Norfolk and Yorkshire. Their children ranged in age and gender (see Table 1). DLD diagnosis was confirmed with the mothers at the beginning of the conversation. Five of the children had known co-occurring diagnoses, the most common being dyslexia and Developmental Coordination Disorder (DCD).

**Table 1:** Characteristics of the mothers interviewed and their children with developmental language disorder

Parent Pseudonym	Child Pseudonym	Age of child	Age of diagnosis	Child gender	Income ^a^	Ethnicity	Number of siblings	Known comorbidities
Corinne	Shawn	6	5	Male	Middle	White Irish	2	DCD, 22q mutation, verbal dyspraxia
Josie	Lucy	6	5	Female	Middle	White British	2	-
Camila	Joshua	6	5	Male	Low	Black British	0	-
Rami	Sera	7	5	Female	Low	White European	1	7q11.23 duplication
Nicola	Abigail	7	6	Female	ND	White British	2	-
Suzanne	Mark	8	5	Male	High	White Irish	1	Dyslexia
Mena	Kevin	9	5	Male	High	White British	1	-
Iona	Pia	9	7	Female	Middle	White British	1	-
Sanne	Elias	9	9	Male	ND	White European	1	-
Jane	Oliver	10	3^c^	Male	ND	White British	1	Dyslexia, APD, traits of PDA^b^
Kenna	Joel	12	5	Male	ND	White British	2	DCD, Dyslexia, Dyscalculia

*DCD = developmental coordination disorder; PDA = pathological demand avoidance; APD = auditory processing disorder; ND = not disclosed.* ^*a*^ *Income groups are defined as ‘Low’ = below £30,000 per annum; ‘Middle’ = £30,000-£60,000 per annum; ‘High’ = above £60,000 per annum .* ^*b*^ *No Autism Spectrum Disorders.* ^*c*^ *Early diagnosis was achieved whilst living in the USA.*

All children, except Pia, had been able to access SLT intervention, either independently (Lucy, Elias, Abigail, Mark) through school (Joel and Joshua), or both (Shawn, Sera, Kevin, Oliver). In addition to this, both Shawn and Mark had received access to occupational therapy intervention. No children had received any specific psychosocial support or intervention; Suzanne and her husband were the only parents to report accessing psychological therapy themselves. Prior to receiving their DLD diagnosis, Oliver and Sera were both diagnosed with glue ear and had grommets fitted and removed. Sera was the only child to be selectively mute (until she was four years old).

For study II, secondary data from a separate study was used. This study explored the challenges and successes of adults with DLD, when reflecting on their childhood and adult lives. Five adults were recruited, between the ages of 31–47 years old (M = 39.4, SD = 5.53). Convenience sampling was used, resulting in a representation of perspectives across three countries in Europe, and two multi-lingual participants. The self-reported language needs and diagnoses of the participants ranged (see Table 2), alongside their occupations (from blue to white collar) and income brackets (low to medium).

**Table 2:** Characteristics of the adults interviewed with developmental language disorder

Adult Pseudonym	Age	Age diagnosed^a^	Gender	Income^b^	Ethnicity	Language needs^c^	Languages spoken at home	Known comorbidities	Relationship status
Farah	40	39	Female	Middle	White European	Very severe	Arabic, English, Spanish	ADHD, Dyslexia, Dyscalculia	Cohabiting
James	47	childhood	Male	Low	White British	DK	English	DCD, dyscalculia	Single
Luisa	37	37	Female	Student	White European	Mild	English, Spanish	ADHD, Dyslexia	Single
Meghan	41	childhood	Female	Low	White British	DK	English	Dyslexia, Anxiety, Depression	Cohabiting
Sammy	31	26	Female	Low	White British	Mild-moderate	English	Anxiety	Cohabiting

*DCD = developmental coordination disorder; ADHD = attention deficit hyperactivity disorder; DK = don’t know;* ^*a*^with language disorder. ^*b*^*Income groups are defined as ‘Low’ = below £30,000 per annum; ‘Middle’ = £30,000-£60,000 per annum; ‘High’ = above £60,000 per annum.* ^*c*^*Self-reported.*

Most adults were able to reflect on their childhood, and the impact their DLD had on them. However, one adult (James) described having no memory prior to when he was 15 years old.

### Theoretical Approach

There was a need to ground the data analysis in the participants’ own perspectives and unique familial worlds; the heterogeneity of samples with DLD (Thomas et al., 2019) heightens the importance of focussing on the individual characteristics of the parents and their children. As a result, interpretative phenomenological analysis (IPA; Eatough & Smith, 2008) was employed for study I, so that any underlying psychosocial patterns could be better understood.

Analysis of data from study II was secondary and conducted using deductive analysis. This approach was chosen such that the interviews were interpreted using the codes developed from study I as a template to build a systematic understanding of the psychosocial experiences of individuals with DLD during childhood (Pearse, 2019). This process took a realist perspective, to reflect the participants’ lived experiences as accurately as possible, and exactly as expressed. As such, it relied less heavily on researcher interpretation, and more heavily on the participant’s own words; as recommended when interviewing individuals with language differences (Goodley, 1996).

### Interview Schedule

A semi-structured interview schedule was used to structure the conversations in both studies (I and II).

For study I, and as recommended by IPA methodology, open-ended and expansive questions were used to allow participants to explore at length their own experiences (Willig, 2017). As such, the interviewer’s main role was to anchor the conversation around eight broad issues, whilst freeing the participant to explore topics and stories they felt were relevant. The eight issues used to anchor the conversation were developed from prior literature on psychosocial difficulties of DLD (e.g. Conti-Ramsden et al., 2019), as well as parenting challenges of children with similar neurodevelopmental disorders (e.g. Woodgate et al., 2008; Moen et al., 2011): (i) a broad introduction to the family set-up and daily life, (ii) their personal journey towards receiving the DLD diagnosis, (iii) the biggest challenges they feel they face in raising their child, (iv) how DLD impacts the relationships within the family, (v) how they and their child cope with these challenges, (vi) how and where they have accessed support, (vii) what they consider to be their child’s strengths, and (viii) their reflections on any need for psychosocial intervention.

Following the principles of public and patient involvement (PPI; Bagley et al., 2016), a parent meeting the criteria for the study was consulted about the appropriateness and clarity of the materials and conversation topics. The interview schedule was then piloted with this parent, ensuring the questions prompted a natural and comfortable dialogue between participant and interviewer. This parent was then consulted again, following the conversation, and suggested no further changes should be made to the schedule.

For study II, the interview was more structured, with emphasis given to providing clarification of the questions asked. This was done through supplying participants with the discussion guide and prompt sheet before the interview, so they had time to prepare beforehand, if preferred. During the interview, the questions were then displayed on the screen, and automatic closed captions were provided throughout. The interview covered six main topics: (i) their journey to diagnosis with DLD, (ii) what they considered their biggest challenges to be, relating to DLD, (iii) how they believe DLD has/has not impacted their interpersonal relationships, (iv) their access to support, (v) their opinions on child psychosocial intervention and (vi) what they consider to be their strengths. For each topic, prompts included reflections on different life stages (i.e. childhood vs. adulthood). A process of PPI to pilot the interview schedule was not used for this part of the study.

### Procedure

Ethical Approval was granted by the Psychology Research Ethics Committee at the University for both studies (Refs:21 − 013, 21–216).

All participants were recruited online, through an advert distributed across the Engage with DLD database (E-DLD; *Engage with DLD*), as well as circulated via Twitter and Mumsnet. The E-DLD database was set-up to connect individuals and families with DLD with researchers; participants self-select to sign up to receive a newsletter promoting different studies (n = 121 parents, n = 13 adults; August 2021). The samples were limited to a small number, such that they could represent individuals from a variety of backgrounds, whilst not over-sampling, which can risk losing the attention required to reflect the nuance of an individual’s experience (Brocki & Wearden, 2006). Purposive sampling was used to ensure that a wide range of life contexts was represented; for example through Facebook groups for ethnic minority or low-income parents.

Once recruited, participants of both studies were required to complete an initial screening questionnaire to ensure they met the required criteria. Parents had to confirm (i) they had a child with DLD between six and 12 years old, and (ii) they were fluent in English. Diagnosis was confirmed through two self-report processes. Due to the variance in nationalities, as well as the different services accessed to receive a diagnosis, no uniform diagnostic report was required. Firstly, parents had to respond to the following questions: “Do you have a child that has difficulties either using spoken language or understanding spoken language?” (answer response “Yes” was required); “Is there a known explanation for this language difficulty (that is NOT DLD/specific language impairment)?” (answer response “No” was required; Bishop et al., 2016). Secondly, the initial part of the interview required parents to confirm the process through which their child was given their DLD diagnosis. A minority of families reported a straight-forward diagnosis process through their local doctor (Kevin, Shawn). For others, diagnosis required multiple self-referrals until the NHS was able to provide the appropriate assessments (Pia, Lucy, Joel, Joshua); or else, resorting to paying for a private assessment (Sera, Abigail, Mark, Elias), especially for those who reported being dismissed due to their families’ bilingualism (Sera, Elias). One child’s family (Oliver) lived locally to a university’s speech and language department, who was able to complete an assessment at an early age.

For study II, adults had to confirm (i) they had a diagnosis of DLD, (ii) they were above the age of 18 years old, and (iii) they were fluent in English (written and spoken). Diagnosis was confirmed through two initial screening questions: “Have you ever received a diagnosis of Developmental Language Disorder, Specific Language Impairment, Receptive Expressive Language Disorder or a different label referring to a primary language difficulty or impairment?” (answer “Yes” required); “Is there a known explanation for this language difficulty? (that is NOT Developmental Language Disorder/Specific Language Impairment)?” (answer “No” required; Bishop et al., 2016).

Following informed consent, all participants were then invited to complete an additional questionnaire to provide basic demographic information. For study I this included: parent and child gender and age, number of siblings, household income, occupation of main income earner, ethnicity and city of residence. For study II this included: age, gender, ethnicity, monthly income, occupation, co-occurring diagnoses, languages spoken at home and country of residence. Answers were not used to exclude participants; all of those who wished to take part were able to. All participants were then interviewed either through a videocall on Microsoft Teams ™, or a phone call, depending on the participant’s preferences. This was audio recorded and transcribed verbatim. Summaries of the interviews, and the overall study results were then shared with the participants themselves (both adults and parents). This minimised the possibility of interpretation bias (Neusar, 2014).

### Researchers’ Perspectives

Reflexivity was of paramount importance during this project. A reflective log was kept by the lead researcher to ensure their years of working in the treatment of anxiety and mood disorders (in neurotypical populations) was not leading to interpretive biases. The remaining authors added expertise from a range of backgrounds specific to language development and health behaviour. This included a senior lecturer in language development and developmental psychology, an associate professor in speech and communication (and speech and language therapist) and a lecturer in behavioural and health psychology. During analysis, presentations were made to a group of clinical psychologists and academics, to ensure that conclusions appeared both fair and grounded in developmental psychopathological theory.

### Data Analysis

Data for study I were analysed following the double-hermeneutic interpretation process, as described by Smith and Osborn (n.d.). As such, the interviewer was considered an active participant in understanding each parent’s perspective on their child, both during the interview, as well as reflectively post-interview. Initial notes were made post-interview to determine any immediate biases the interviewer may hold, before the transcripts were then read and re-read independently by both the interviewer and an additional researcher. Developed themes were listed by both individuals independently, before meeting to discuss thematic clustering and the development of superordinate themes. The transcripts were frequently referenced to ensure themes remained reflective of the original conversations.

Further supervision was then held with additional academic psychologists and an academic speech and language therapist where overall themes were discussed. These themes were then shared with four of the parents who were interviewed, to ensure they were happy with the conclusions drawn from the conversations. All four were happy with how the results reflected their experiences.

Data from study II was analysed thematically, using the codes from study I as a template from which to review the data. Stages in analysis included: familiarisation with each interview, coding of data against the study I coding template, pooling themes across interviews, and reviewing the evidence by focussing on the participants’ direct quotes (Goodley, 1996). Summaries of all conversations were shared with the adults who were interviewed, all of whom reported being comfortable with the way their stories had been represented.

## Results

**Fig. 1:**
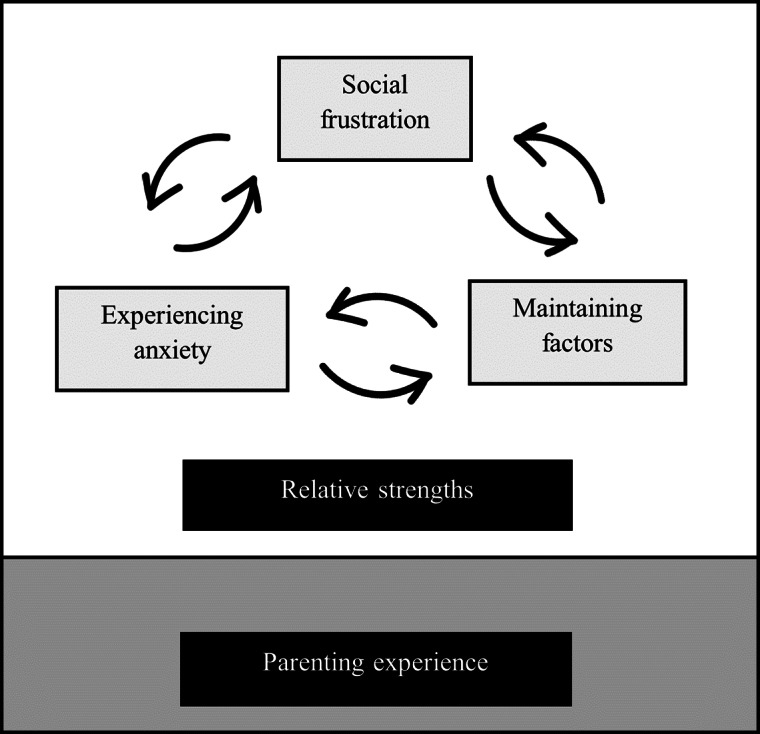
Visualisation of the core themes identified by mothers through a process of interpretive phenomenological analysis, including an ongoing maintenance cycle of psychosocial priorities: experiencing anxiety, social frustration and maintaining factors

Five overall themes were developed from study I, each of which contained three separate sub-themes (see Fig. 1). Three of these overall themes are conceptualised as a maintenance cycle, each contributing to one another and exacerbating the child’s difficulties: experiencing anxiety, social frustration and maintaining factors. Whilst recognising these themes, attention must be given to the difficult and isolating circumstances both parent and child navigate on a daily basis. No parent placed any blame on their child for the challenges they described. Furthermore, three overall relative strengths of children with DLD were also developed from data in study I, and fully supported by data in study II. Experiences of parenting a child with DLD are explored in a separate theme.

**Table 3:** Themes developed from analysis of studies I and II.

Theme	Description	Supporting quotes
**Experiencing anxiety**	Anxiety was described by both mothers and adults with DLD as manifesting in both cognitive and behavioural symptoms.	*“I have anxiety and depression because of the way that life has made me feel […] constantly worrying about different things all the time.”* (Meghan, 41 years old)
Intolerance of uncertainty	Children were described as exhibiting control-like behaviours, such as preference for sameness and reliance on routine. Such behaviours were understood to be a way to cope with anxiety. Adults with DLD reflected on finding routine similarly comforting.	*“He was very kind of routine based […] he needs to understand in whatever he’s doing what the kind of parameters are or what the expectations are […] he just needs to understand what’s expected of him”* (Jane, about Oliver, 10 years old)
Rumination	Cognitively, many children and adults struggled with managing their worries. Adults were able to describe experiencing ongoing loops of negative thoughts, characteristic of anxiety.	*“He had a sick bug like one to two years ago and he did not like it, so now being sick is a big worry: ‘what if I’m sick’, ‘well if you are, you’ll be fine’ […] now if he gets unpleasant sensations in his tummy he’s like ‘I must not eat cause I might be sick’ and I’m like ‘but I think that’s hunger’”* (Mena, about Kevin, 9 years old)
Negative interpretation bias	Individuals with DLD were described as having a tendency to interpret ambiguous situations negatively. This seemed particularly heightened during social ambiguity.	*“Hyper paranoid about what people think […] you can see the body language and you can see if someone looking awkward […] and maybe assume the worst […] there’s definite social anxiety”* (Sammy, 31 years old)
**Social frustration**	All adults and many children experienced frustration during social interaction, brought on by either the behaviour of others, or more directly related to their language differences.	*“[I would] hand on my own quite a bit[…] I have experienced bullying as a child[…] you may not know exactly what they are saying[…] it just exacerbates social anxiety”* (Sammy, 31 years old)
Misunderstanding others	Many children were described by their mothers as struggling to understand the intentions of others, more so than their typically developing peers and siblings. This caused problems with both misunderstanding interactions, as well as engaging with individuals who do not have their best interests at heart.	*“When someone tries to help her, she doesn’t always see it as help and pushes people away”* (Nicola, about Abigail, 7 years old)
Social exclusion	Parents described watching their child be actively excluded from play opportunities by their peers; adults with DLD remembered not only being ‘left out’, but being more actively bullied.	*“She’d find it so hard to structure what she wanted to say it’d be so long winded and really hard to understand that […] I’ve seen it with kids you know they’ll just go off and play with something else […] she’s never been invited anywhere after school”* (Nicola, about Abigail, 7 years old)
Social withdrawal	It was common in childhood for those with DLD to seek-out solo play. Although parents of children with DLD were uncertain of the motivation behind this, adults with DLD reflected on how this was a preference, away from the difficulties of interacting with their peers.	*“I had a lot of fun with myself […] I think that was a bit making me bubble up. I didn’t care what others were thinking about me […] when I was alone, I was super happy.”* (Farah, 40 years old)
**Maintaining factors**	Specific characteristics were consistently agreed as contributing to the maintenance of anxiety and social frustrations.	*“Part of [the meltdown] might have been triggered by sheer exhaustion because he is needs it takes so much more of his energy to get through a day at school than other typical child[ren] […] I think that was a major thing during the past six months that lead to this huge panic attack”* (Sanne, about Elias, 9 years old)
Lack of emotional regulation and understanding	Both parents and adults described difficulty in expressing emotions during childhood, often resulting in acute emotional outbursts and misunderstandings.	*“I was not able to express myself really […] I might have done it through drawing […] I think was a way to, you know, filter the emotions and help me to calm down”* (Luisa, 37 years old)
Exhaustion	Parents described how children were completely exhausted after the school day, having had to work so much harder than their peers to follow what was required of them.	*“She is so overwhelmed […] she has to work so hard when she’s in school to understand things to know what she’s meant to be doing”* (Josie, about Lucy, 6 years old)
Low self-esteem	Difficulties with low self-esteem were reported to start in childhood and continue to adulthood. Contributing factors were children’s negative academic comparison of themselves with their peers, as well as recalling other children’s hurtful comments.	*“[I remember] not feeling as clever [as] anyone and not being able to do things other people can do. Seeing people getting the praise for doing like exams and things […] It really pulled me down like I’m not good enough […] It knocked my confidence a lot”* (Meghan, 41 years old)
**Relative strengths**	There were interpersonal and intrapersonal skills that children with DLD excelled in, relative to neurotypical children; either observed by parents, or recalled by adults.	*“I started to see the metaphors and the links between ideas in a way that other people just didn’t get[…] you can kind of use your visual skills that are like heightened in DLD.”* (Sammy, 31 years old)
Empathy and kindness	Some adults with DLD believed they had learnt to have a heightened perception of other’s body language and therefore mental states, due to being able to rely on direct language less. Parents described this ability in their children, alongside their drive to care for others, and make sure they are happy and comfortable.	*“He’s definitely empathic about some things. He doesn’t always let you know about it, but he’s let’s say he’s very observant of people and animals, if he finds a moment to do so, and then you are surprised by how much he will have taken in”* (Sanne, about Elias, 9 years old)
Creativity	Creativity was described for many through an enjoyment of drawing, as well as performance or make-up. It was described as a non-linguistic form of expression.	*I use my eyes, it’s my biggest tool […] naturally I would do drawing a lot. I like to draw a lot. I was spending a lot of hours drawing.* (Farah, 40 years old)
Resilience	Parents spoke with great pride about how resilient their children were, that despite the hardship and set backs of everyday life, their children continued to try the next day.	*“No matter what happens to her she’s so resilient, she’s just constantly happy whatever circumstances […] she just brushes it off ‘oh well you know tomorrow we’ll start again’”* (Rami, about Sera, 7 years old)
**Parenting experience**	There were three unanimous experiences described by all parents, all described as being worsened due to the lack of awareness of DLD.	*“It is incredibly sort of isolating […] it’s just it’s not always as easy as sort of parenting another child and it’s full of worry.”* (Jane, parent of Oliver, 10 years old)
Isolation	Parents felt very unsupported from the moment of diagnosis, and felt they had little support networks to rely on (professionally and personally).	*“We were never sat down and explained ‘okay this is the disorder, this is how it’s likely to impact him’ I mean it’s been assumed that I would find that out on my own”* (Corinne, parent of Shawn, 6 years old)
Stress	Descriptions of parenting stress included feeling the need to be hypervigilant over their child to protect them. Parents described exhausting themselves with constant crisis-planning in order to calm their worries about their child’s wellbeing.	*“I would’ve pushed myself quite probably to burn out, do you know, in terms of trying to read things, and how come I’ve never heard of this before, and what can we do to help our son, you know, and I would’ve [been] up late at night you know in a job that was quite intense anyways so I suppose burning the candle at both ends”* (Suzanne, parent of Mark, 8 years old)
Concern over the future	Parents were hugely concerned over their children’s futures: what level of independence they might be able to achieve. The lack of representation of adults with DLD created a huge level of uncertainty.	*“When we’re seventy five will [Lucy] still need to be living with us or […] will she be able to have a job […] I can remember lying awake one night worrying about it”* (Josie, parent of Lucy, 6 years old)

### Experiencing Anxiety

The experience of anxiety came across in different ways for different children. Each mother associated a separate set of behaviours with each of their children’s experience of anxiety, from nail biting to constant worrying. This was echoed by the adults with DLD; describing behaviours such as thumb sucking and hiding behind parents as linked to constantly feeling *“hypervigilant and hyperalert”* (Sammy, adult). Across all participants, three main characteristics of anxiety was found to be most common:

#### Intolerance of Uncertainty

Many children appeared to experience anxiety as a response to uncertainty. This was expressed primarily by those who were described as ‘thriving’ on routine. It was common for the whole family’s weekly schedule to revolve around the child’s need for predictability; many described using big wall-calendars, with pictures of places they’re going to that week pinned up (parents: Suzanne, Josie, Camila, Kenna).

*“He’ll look in the morning and see what we’re doing, or if it’s the holidays we write when he has different things […] there’s been a couple of times I’ve had to change what we’re doing after school and he gets very upset and takes that very personally”* (Camila, parent).

Sammy (adult) remembered finding routines *“comforting”*, and mothers explained how, for their children, ‘*meltdowns*’ felt inevitable if they did not know plans well in advance. It appeared as though these coping mechanisms gave children a sense of control, allowing them to mentally prepare beforehand:

*“[Joel] will have planned out in his head how something is going to pan out and if that changes then he finds that really difficult because that isn’t a circumstance that he’s anticipated and then he’s not prepared for it so he would find that really stressful”* (Kenna, parent).

People are also not allowed in Joel’s room or to touch his things, which suggested to his mother that he likes to have things a certain way. Several mothers described similar behaviours as *“OCD”*, referring to their child’s need for tidiness and ultimately helped them *“feel more secure”* (Josie, parent). For example, Nicola (parent) describes Abigail’s tidiness as *“a bit OCD”*. She believes this is Abigail’s way of gaining control when she usually feels out of control:

*“She gets quite cross so if I put something on her desk like her clothes for the next day she’ll move them cause it’s she likes things to be in a certain way […] her room used to be quite messy but she literally threw everything out and everything’s organised and I said to my husband ‘I think that’s her way of being in control of something’”* (Nicola, parent).

Additionally, some children found comfort in a preference for sameness, similarly providing them with a sense of control and familiarity. For example, Lucy (child) likes to eat from the same white bowl every morning and be the first one downstairs. If neither of these things happen, Lucy is described as getting incredibly anxious and upset, taking her mother Josie a long time to reassure her and calm her down. Similarly, Iona and Mena (parents) despair at their children’s *“disastrous”* and strict eating rules regarding how much they will eat and of what:

*“She has two Weetabix and every morning she has to leave one spoonful […] and I’m like ‘you can’t, stop doing that’ and she’s like ‘but I like to leave one spoonful’ so every morning she has to leave one spoonful […] that’s the most difficult part I think, you’re having to make different things because she won’t eat anything […] I’ve resulted in just buying her vitamins”* (Iona, parent).

Some mothers expressed worries around the unknown more verbally. Sera (child) worries about new places she has not visited before and new people she does not know, which, for example, impacts hospital appointments, as she will not let people near her:

*“We would have to go into a room and I would say [to the healthcare professionals] ‘can you give her ten minutes’ so she does probably, I think she needs to observe first of all to see ‘what’s around me, am I comfortable with this’ and then she would be much better”* (Rami, parent).

Children had different coping mechanisms for this underlying anxiety. For some children (Henry, Kevin and Sera) and adults with DLD (Sammy), this fear of the unknown made them very attached to their maternal figures, relying on them being present to provide comfort:

*“He won’t do anything if I’m not right there […] about five times a day I’ll go ‘remember Kevin, who’s got your back’ and he’ll be like ‘you do mum’ like I had to peel him off me at the bus stop this morning”* (Mena, parent).

Camila (parent) described how the only way to get Joshua to stop “*constantly touching*” her is to *“hold and fiddle with”* these specific small toys (e.g. fidgets) he has, that seem to *“settle”* him. This habit was also mentioned by Sammy, who liked *“really soft things like feathers, by using or like self-soothing”* when she was young.

#### Rumination

For children who are older and have developed more verbal fluency (Elias, Joel, Kevin, Oliver and Pia), expressing worries over future situations and ruminating over past negative experiences was extremely common. Sometimes this rumination occurred as a more direct result of the difficulties the child’s language disorder presents. For example, Oliver (child) is very preoccupied by “*being good”* and understanding what is expected of him in different situations, so that he can *“do the right thing”*. His mother believes this has stemmed from being told off in class for not appearing to have been listening, or generally feeling lost as to what he is required to do. Nonetheless, he requires constant reassurance.

In other cases, rumination appears to stem from past experiences that the child may not have been able to either fully process or understand at the time. As such, Kevin (child) has a strong preoccupation with being ill, and an ongoing fear when preparing for his fortnightly stay at his father’s. His mother believes this stems from an incident when he was very ill when visiting his father. Similarly, anytime he is particularly hungry, he often panics that he is ill again, confusing the sensation with nausea. The only thing that appears to be able to settle him is recording videos of himself each time he visits his father, reassuring himself that nothing bad happened and that next time he need not worry so much.

*“He had a sick bug like one to two years ago and he did not like it, so now being sick is a big worry: ‘what if I’m sick’, ‘well if you are, you’ll be fine’ […] now if he gets unpleasant sensations in his tummy he’s like ‘I must not eat cause I might be sick’ and I’m like ‘but I think that’s hunger you’re feeling so you must eat or you will be sick’* (Mena, parent).

Generalised ‘overthinking’ was also mentioned by both Elias’ mother (Sanne), as well as by adults Farah and Meghan. Sanne believes that Elias’ late diagnosis of DLD (9 years old) contributed to the internalisation of his difficulties in this way. Unable to access appropriate support, he suffered numerous panic attacks, one of which resulted in hospitalisation. Although his panic attacks have stopped since receiving both the diagnosis and more language support, he still requires constant reassurance and *“praise for a lot of tiny things”*. Similarly, Farah (adult) believes if she had received a diagnosis earlier (than 39 years old), she wouldn’t have carried so much anxiety with her through childhood. Nonetheless, despite receiving support from a young age, Meghan (adult) described how she was “*constantly worrying about different things all the time. What happens if this happened? What happens if this happens?”;* an experience that has followed her through to adulthood.

#### Negative Interpretation Bias

Children seemed to have a level of social *“paranoia”* (Sanne, parent), led by their tendency to negatively interpret innocent situations. In fact, Sammy (adult) described how she gets “*hyper-paranoid about what people think about [me]*”. Camila (parent) described with great humour how Joshua can take even the most mundane situations incredibly personally. There appeared to be a particularly heightened sensitivity to social rejection or ridicule, where not considered a rational response. For example, both Kevin and Pia were described as being concerned that when someone is laughing, they are the ones being laughed at.

*“When we were looking back at old videos on my phone, if there was something that I found funny about a video that she was in I would laugh and she would tell me to stop laughing she’s like ‘why are you laughing at me, stop laughing at me’”* (Iona, parent).

### Social Frustration

Due to their language difficulties, it was common for children to experience frustration when interacting with others socially. Misunderstanding the intentions of others often led to children reacting inappropriately, sometimes resulting in unnecessary conflict. As described by parents and adults alike, the lack of patience and understanding demonstrated by most of their neurotypical peers also resulted in either active exclusion of the child with DLD, or that child withdrawing themselves from play.

#### Misunderstanding Others

Many mothers spoke about how their children struggled to understand nuanced social situations. Some mothers refer to their own fears around their child’s safety, when their child struggles to understand the difference between being kind to others, and the concept of ‘stranger danger’:

*“She’s no awareness [that] she shouldn’t be talking to people she doesn’t know […] this lady had a dog and [Pia said] ‘You can have me in your caravan and I’ll play with your dog’ […] it doesn’t matter how many times you talk to her about [stranger danger], she just can’t retain that information”.* (Iona, parent).

Children were also described as being a little ‘out of sync’ with their peers, often misunderstanding someone’s intentions and struggling with cooperation. For example, Nicola (parent) described how this can often be the source of arguments between Abigail and her siblings:

“*I was watching her and her brother yesterday on the trampoline and she’d hurt her foot and he was trying to help her but she doesn’t always see people as helping her […] he was being really gentle and kind and trying to look at her foot and then she’s like ‘ohh get away get away’ and then kicks [him]”* (Nicola, parent).

Although none of the adults recalled misunderstanding intentions as children, Meghan (adult) described how she had been taken advantage of by a romantic partner as an adult, who stole a large sum of money off her before she realised she was being manipulated.

#### Social Exclusion

Children were often excluded from social gatherings by their peers; one mother even described how her son had not been invited to a birthday party in over a year. This seemed to stem from their difficulty in ‘fitting in’, either appearing much quieter, or standing out because they do not understand the social nuances of appropriate interaction.

“*I think [Joel] ends up basically just annoying people and then they kind of distance themselves from him and he finds that really hard […] he feels really rejected by it, but equally he can’t control his impulse not to sort of keep going at them and badgering them to do something or talk to him”* (Kenna, parent).

Mothers were understandably the most concerned when their children had few friends. They spoke of how frustrating this was, as their children needed the social interaction practice more than neurotypical children. This often increased parent’s hypervigilance over their children in these social situations. Mothers described watching their children struggle to articulate themselves (“*it’d be so long winded and really hard to understand”;* Nicola, parent), and how their peers would just walk away and play with someone else rather than wait or help them out.

This was supported by accounts from the adults with DLD (adults: Meghan, Farah and Sammy). Whilst Meghan described being “*kind of completely left [out*]”, Farah and Sammy experienced more active bullying, recalling how children made “*me believe that I was stupid*” (Farah, adult). Sammy described this as a particular issue in primary school, and how later on she found solace in “*quite [a] nice group of friends that were the outsiders*”.

#### Social Withdrawal

Some children found comfort in solo play, however mothers were aware that this only reduced their ability to practice social communication. Jane described watching Oliver interact with other children for a few moments before taking himself to play on his own. Some children had tactics in order to avoid conversation; whilst some children used ‘silliness’ and physical play to distract from the conversation, Oliver was described as ‘*shielding*’ himself from participating in class, by pretending to be very grumpy when he arrives at school.

“*I think it’s a sort of protection thing so he’s not expected to respond or participate […] he tries to come across as really unapproachable so people will leave him alone.”* (Jane, parent).

This was an incredibly common memory amongst the adults with DLD. Both Luisa and Farah (adults) described how being *“quiet in the classroom”* (Luisa, adult) helped them feel as though they could “*hide*” their difficulties. Farah particularly described how she felt her solo-play gave her a protective “bubble”:

*“You are always too afraid like […] maybe they discover I’m not good enough to be here […]I think my experience is what protect me from all that. I was having a lot of fantasy imagination. I had a lot of fun with myself […] when I was alone, I was super happy.”* (Farah, adult).

Josie raised an interesting insight into her daughter’s solo play not necessarily being ‘*an active choice’*:

“*She wouldn’t necessarily use the word lonely, but you know like if there’s someone in a picture on their own, she would be able to say like ‘they want a friend’ […] ‘they’re on their own, they’re sad’”* (Josie, parent).

This association between lone-play and sadness perhaps reveals Lucy’s preference to be playing with children, and therefore that lone play is a choice primarily born from ease. Similarly, Sammy (adult) described how she would “*hang on [her] own”* due to her shyness, but watch others interact, which she felt only exacerbated her social anxiety:

*“You may not exactly know what they’re saying […] but you can see the body language and you can see if someone [is] looking awkward or not, and maybe assume the worst […] in my early experiences, I think [I] was just minding my own business”* (Sammy, adult).

The drive to socialise was more easily realised when interacting with other children with similar language levels to them: those with special educational needs (children: Lucy, Sera, Joel and Connor), or younger children (Abigail, child):

*“When [Abigail] was at preschool she always used to play with like the babies and the younger ones […] I think because they were sort of at a language similar to her she felt safe”* (Nicola, parent).

Similarly, Sammy (adult) remembered with great fondness how she was given the role as a “buddy” for younger children at school:

*“My last year, [aged 11], I did form friendships with slightly younger children… being like a buddy for like a younger child… I could form so much better friendships with them… [some] of the best friendships I’ve had because I felt like I wasn’t going to be condescended to”* (Sammy, adult).

### Maintaining Factors

There were a number of characteristics that exacerbated the aforementioned anxiety and social difficulties. These either appeared as secondary effects of having DLD, such as exhaustion from social contact and low self-esteem, or as co-occurring difficulties, such as difficulties with emotion regulation.

#### Difficulties with Emotional Regulation and Understanding

Experiencing difficulties with emotion regulation was emphasised as a key worry for most mothers. It was described how their children progressed quickly and unpredictably through different emotions, which were often expressed very physically, making it challenging for those around them to know how to respond.

“*She gets really cross, sort of stamps her feet, […] sometimes growls […] her fists clench […] and then yeah then the next minute it’s almost like a witch’s cackle and then she’s back to the being cross again. It sort of flips […] she’s very up and down, she’s hard work”* (Nicola, parent).

Sanne (parent) described how Elias was more “*reactive”* than other children and his siblings, often as a result of misinterpreting social interaction, or being misunderstood himself. Similarly, children were described as becoming increasingly quick to anger with age, which worried mothers as their child becomes larger, stronger and perhaps more aggressive.

Children’s difficulties in their own emotions was linked by some mothers to their difficulties self-regulating. Josie (parent) described how it often helps calm Lucy down to sit with her and explain what has just happened and why she is upset. Similarly, Lucy was described as having trouble linking emotions across time.

“*She finds transitions quite hard so you could’ve been out had a really nice day she we’ll be in the van going home and we’d say ‘did you have a nice time’ and she will say ‘no’ […] I think it’s because she’s like not able to separate ‘I feel really sad now we’ve had to finish doing that’ from ‘I really enjoyed doing it’”* (Josie, parent).

Luisa (adult) also recalled struggling to express her emotions, and relied heavily on drawing as her “*escape*” from her emotions:

*“I was not able to express myself really […] I might have done it through drawing. I used to do lots of drawing and painting […] You know, you just don’t have to think, you just do it. So those sorts of things helped to get myself into my own bubble and forget about everything […] I think was a way to, you know, filter the emotions and help me to calm down”* (Luisa, adult).

Only Sera and Mark (children) were described as having good emotional understanding and regulation. Interestingly both of their mothers had clear hypotheses as to why this might be the case. Sera’s mother gives credit to Sera’s use of sign language when she was younger, as an alternative method for understanding and expressing her emotions. In contrast, Mark’s mother referred to Picture Exchange Communication System cards as “*game changing”*, providing him with visual prompts as further context for emotions.

#### Exhaustion

Given the difficulties in communicating, the mothers all described a certain level of exhaustion felt by the children, particularly at the end of the school day. This exhaustion seems to result in outpouring of emotions on their way home, as *“it takes so much more energy to get through the school day”* (Sanne, parent):

*“When she’s picked up from school she is in complete shutdown or meltdown […] it’s just because she is so overwhelmed […] she has to work so hard when she’s in school to understand things to know what she’s meant to be doing”* (Josie, parent).

Luisa (adult) shed some light on this, and how she felt she had to work so much harder than her classmates:

*“I tried so hard […] I spent hours of days studying and really memorising […] I used to summarize the chapter and copy, copy, copy, copy, copy. Until I memorise everything, it was the only way for me. To get it done [and], you know, to sit in our exam and pass […] [I] remember, my best friend only like listening during the lessons. And then maybe she will go over my notes. She will get better marks on me. I don’t like how.”* (Luisa, adult).

The overstimulation throughout the day was described as having two possible impacts on the children’s sleep. Some slept so deeply at night that bed wetting was frequent, barely stirring whilst their mothers changed the sheets. Alternatively, Iona and Kenna (parents) described how their children struggled to wind down. Pia (child) could be found wandering the corridors late at night *“worrying”* about different things that may happen, thus exacerbating the tiredness further:

*“She just can’t turn her brain off and go to sleep that she sits and thinks things over and over ‘cause she worries quite a lot […] about things that don’t affect her so like the school meal was changing but she eats bread and butter every day [instead]”* (Iona, parent).

#### Low-self-esteem

For older children who had grown more aware of their difficulties, self-esteem emerged as a key concern for mothers. These concerns spanned both academic and social confidence, where children worried that others might think they are stupid or not like them as a result. This can lead to children giving up on tasks quicker than necessary, concluding that they cannot do it because they are ‘stupid’. This is not helped by the fact that children at school comment on their differences: *“[Joshua] told me that somebody called him stupid before and, oh my God, broke my heart”* (Camila, parent). As a result, some mothers described working tirelessly to provide their child with reassurance and support to improve their confidence: *“I work my little socks off making sure his self-esteem [is secure] […] he’s got a sign on his wall that’s like ‘I am brave I am fearless’ and he just looks at that over and over and over”* (Mena, parent).

Low self-esteem was certainly something the adults with DLD recalled from childhood, as well as an issue they continued to struggle with as adults. Farah (adult) described how, although she knows she’s smart, she will *“always have that confidence problem”*, which she felt stemmed from other people’s behaviour towards her. She describes being mocked, not only other students, but teachers too, who *“were laughing at my face in front of class”* for struggling to keep up. For Meghan (adult), it was the awareness of her differences that she felt impacted her confidence:

*“[I remember] not feeling as clever [as] anyone and not being able to do things other people can do. Seeing people getting the praise for doing like exams and things, wishing you could be like that […] It really pulled me down like I’m not good enough […] It knocked my confidence a lot”* (Meghan, adult)

Interestingly, some children had learnt various masking behaviours to appear more confident than they are, but not to any great social success:

“*He hasn’t got any self-confidence but then he’s got lots of kind of externalised behaviours that would make you think that he has but it’s a show […] So, if he saw like a group of children his age in the park he would kind of put on like this really exaggerated walk or say something really loudly to make out like he was like really confident and really cool but […] he’s not kind of sophisticated enough to pull that off”* (Kenna, parent).

### Relative Strengths

Mothers spoke enthusiastically about their children’s strengths. Empathy and kindness were displayed through the children’s thoughtful and caring actions, when not always able to articulate themselves through kind words. Creativity appeared to be a non-verbal way of expressing themselves, and mothers remained incredibly impressed by their children’s resilience and determination to keep trying every day, despite the frequent set-backs.

#### Empathy and Kindness

All children seemed to have kind intentions, even when their social skills did not allow them to effectively convey this. In some, there was particular care given to animals, for others it was family members who received special attention:

*“I was sick the other week I just wasn’t well and she wanted to make sure that I was alright she kept bringing me in water and she wanted to lie beside me and make sure that I was okay”* (Iona, parent).

In some instances, children seemed inclined to ensure everyone else felt included in social activities, for example being the first child to welcome in a new student:

*“He was the one who approached the new guy a year ago …I got a message by the teachers pointing out that they thought that was really great […] he’s definitely empathic about some things. He doesn’t always let you know about it, but he’s let’s say he’s very observant of people and animals, if he finds a moment to do so, and then you are surprised by how much he will have taken in”* (Sanne, parent).

Adults with DLD described how they had learnt to be more *“hypersensitive to other people’s physical bodily states”* (Sammy, adult) and body language growing up, because they could not rely on language as easily to *“understand a situation”*. Sammy believed this contributed to her ability to be *“highly empathetic”*, describing: “*I can fully read a lot of people quite well, emotionally speaking, and understand the situation*”.

#### Creativity

Creativity was an interesting, shared strength that most of the children exhibited. Some were described as fantastic performers (dance or acting) and others very artistic (drawing or make-up). Camila (parent) described how Joshua really thrived in these moments: ‘*centre stage loving life’*. Art and performance appeared to be a way for him to express himself without language, and derived great joy from it. Elias (child) was described as creating long and detailed comic strips of social scenes, to great effect.

“*He really shows the mood and thoughts of the little, tiny people […] he’s actually just started a proper art class […] after one session the teacher sent me a message that he is by far the best student…even though he can sometimes not necessarily put all the words to it correctly, it might be expressing the rollercoaster that’s going on inside himself”* (Sanne, parent).

As mentioned, Luisa (adult) described drawing as a “*escape*” for her emotions and identifies as having “*always been very creative*”. Farah (adult) also described herself as *“spending a lot of hours drawing”* as a child and believes her eyes are her *“biggest tool”*. For this reason, she always preferred visual subjects and activities:

*“For example, history, I was a terrible, terrible, but in art history I was really good […] history [of] art [was] perfect because yeah, if you have to talk about art, [you are] having in your head a painting […] [if] I can’t visualise it, I don’t know what to do”* (Farah, adult).

Importantly, both Luisa and Farah have ended up using their creativity in their occupations: fine art and mechanical design.

#### Resilience

All mothers described great admiration for their children, that despite their difficulties, they persevered and showed great resilience. Corinne (parent) described how her son Shawn has a brilliant way of finding alternative ways of doing things or tackling a problem. It was not uncommon for mothers to describe how watching their children was a good reminder to be more patient, adaptable and resilient themselves.

*“No matter what happens to her she’s so resilient, she’s just constantly happy whatever circumstances like you know she has so many hospital appointments she just brushes it off ‘oh well you know tomorrow we’ll start again’ […] she just gets on with things” (Rami, parent).*

### Parenting Experience

The experience of raising a child with DLD involved many shared difficulties, seemingly regardless of where the family lived, and therefore the different public services they had access to. All mothers felt isolated due to the lack of awareness and support provided, resulting in a high level of stress. Similarly, the lack of guidance around what the future may hold for their child meant mothers remained uncertain and fearful over what further challenges they should anticipate.

#### Isolation

One shared experience of all mothers was the amount of isolation they experienced from the point of their child’s diagnosis. Regardless of background, only one parent had heard of DLD before (Nicola, parent), and they all felt they were left to their own devices to figure out both the prognosis, as well as the level of support their child needed. As a result, and the lack of awareness amongst their peers, all mothers felt completely lost, relying on random websites to provide them with basic information. Many had turned to social media groups to try and get advice from other mothers and seek out some form of community.

*“I’ve had to look at these forums and things like that to find, you know, nobody said to me because Shawn has DLD he’s more at risk of dyslexia, I found that out myself online. […] We were never sat down and explained ‘okay this is the disorder, this is how it’s likely to impact him’ I mean it’s been assumed that I would find that out on my own”* (Corinne, parent).

#### Stress

Understandably, stress was an equally common experience amongst mothers. With little help and *“no support”* (Suzanne, parent), mothers felt like it was their responsibility to both advocate for their child, as well as overprepare for any challenges that may lie ahead. Hypervigilance over their child was one of the main responses to this level of stress, leaving mothers feeling exhausted, constantly having to *“think one step ahead”* (Sanne, parent):

*“I would’ve pushed myself quite probably to burn out, do you know, in terms of trying to read things, and how come I’ve never heard of this before, and what can we do to help our son, you know, and I would’ve [been] up late at night you know in a job that was quite intense anyways so I suppose burning the candle at both ends” (*Suzanne, parent).

#### Concern Over the Future

The lack of representation of DLD in adulthood was hugely concerning for mothers. It created both a fear and uncertainty over what might be possible for their child once they grow up, from relationships to careers. Without any *“positive adult role models”* (Josie, parent), mothers were left fearful that their children might not be able to live a fully independent life:

*“I love my children and obviously now I would like them to live with me forever but ultimately you want to raise them to be independent, happy, successful adults […] When we’re seventy five will [Lucy] still need to be living with us or […] will she be able to have a job […] I can remember lying awake one night worrying about it and like deciding that the best solution was that we needed to have another child [to look after Lucy after we die], because Lucy’s the youngest I was like telling [my husband] the next morning he was like ‘you’ve just gone mad now haven’t you’”* (Josie, parent).

Conversations about careers and having families of their own became common sources of further stress for the mothers, as they were left unsure whether or not to manage their child’s expectations:

“*She’s talking about having babies and getting married […] it’s just things like that where you think ‘god it’s so beautiful that you want to be all that when you’re older but how are you gonna cope with it’”* (Iona, parent).

## Discussion

This study has provided new insights into an aspect of DLD that is both under-researched and under-resourced: the psychosocial difficulties of children with DLD. Interviewing children’s mothers provided an opportunity for a more in-depth understanding of the manifestation of these difficulties and gain an insight into the intervention priorities of the family. Additional data provided through the childhood memories of adults with DLD provided first-person validation of four overall childhood themes: experiencing anxiety, social frustration, maintaining factors and relative strengths. Parent’s unique experiences were represented in a fifth and final theme: parenting experience.

The children’s experience of anxiety was identified as a key concern, described by mothers through different observed behaviours (e.g. worrying and nail biting). This builds on previous literature that demonstrates high rates of anxiety amongst children with DLD (Botting et al., 2016), and suggests that this anxiety could underpin other, less well documented, behaviours. For example, many mothers described routined behaviour and preference for predictability, which were understood to be their child’s way of coping with an intolerance of uncertainty. Intolerance of uncertainty is a well-established characteristic of anxiety (Carleton, 2014) and has been suggested to be a mediating link between routined behaviours and anxiety in children with autism (Boulter et al., 2013; Joyce et al., 2017; Stark et al., 2021); routined behaviours help the children cope with the uncertainty (Rodgers et al., 2012). Previous qualitative work has suggested children with language disorders experience uncertainty in everyday social situations (Lloyd-Esenkaya et al., 2021). Whilst recent psychosocial interventions have begun targeting the management of this perceived uncertainty amongst those with DLD (e.g. Ward & Galpin, 2021), there remains little understanding of why this relationship between DLD and uncertainty exists. More research is warranted, to understand the manifestation of such intolerances, and determine whether interventions used for ASD may be relevant for children with DLD (e.g. Rodgers et al., 2022).

The children also exhibited negative interpretation biases, which related particularly to anxiety around social situations and other people’s opinions of them (a core characteristic of social anxiety; Weeks et al., 2009). Despite these children not presenting with depression yet, based on prominent cognitive models (Disner et al., 2011) and depression’s co-occurrence with social anxiety (Weeks et al., 2009), the maintenance of these negative cognitive appraisals into adolescence could contribute to the development of depression. This has been documented in studies of older children (Botting et al., 2016), particularly when paired with the low self-esteem described in the older children in this sample.

More insight was provided into the potential motivations behind social withdrawal, a behaviour that has been described as common in DLD populations (Lloyd-Esenkaya et al., 2020). Mothers often viewed this withdrawal as a self-preservation tactic, for example allowing the children to avoid anxiety provoking situations such as a conversations, out of fear that others might think they are ‘stupid’. Fear of negative social evaluation is a core characteristic of social anxiety (Leary et al., 1988), and can lead to a more submissive confrontational style. This suggests that these early cognitive patterns and maladaptive coping mechanisms could contribute to the development of more complex social difficulties later in life (Durkin & Conti-Ramsden, 2010; Whitehouse et al., 2009), and therefore provides an interesting focus for future intervention.

Interestingly, mothers appeared far less concerned about their children’s potential externalising symptoms, such as tantrums and hyperactivity. This is in contrast to quantitative research that has drawn attention to the high rates of externalising issues amongst children of this age with DLD (Yew & O’Kearney, 2013). What the current study instead suggests, is that mothers are more concerned with the social and emotional ‘triggers’ that they observe as causing the externalising behaviours. For example, many connected tantrums to emotional dysregulation, or understood physical aggression as resulting from social misunderstanding. As such, these mothers appeared more focussed on getting help for what they understood to be the underlying socio-emotional causes, rather than the behavioural difficulties themselves. There are many cognitive and theoretical models that could defend these assumptions (Leland, 2014), however they have not yet been explored in DLD populations.

As previously suggested by van den Bedem (2020), emotion regulation appeared to play a key role in the escalation of situations into what mothers described as ‘meltdowns’. From mothers’ perspectives this was a key difference between the children with DLD and their neurotypical siblings. The stories told suggest emotion regulation plays a role in fuelling heightened emotional experiences, further complicating social interactions and potentially exacerbating the anxiety; a pattern also documented in research amongst children with autism (Vasa et al., 2018). Suggestions were also made that the ability to express emotions through other means in early childhood (e.g. sign language) could help prevent the development of emotion regulation difficulties. This is not only supported by previous evidence of the relationship between the ability to express oneself and the development of emotion regulation strategies (e.g. Cole et al., 2009), but provides an area for potential intervention.

The high level of isolation and stress expressed by all mothers in this sample is concerning, given the positive association between parent wellbeing and child psychosocial outcomes (Albanese et al., 2019; Masarik & Conger, 2017). It supports other qualitative studies that guidance provided to parents is insufficient and can cause unnecessary added stress (Ash et al., 2020) and suggests the provision of support by the UK heath care system has not improved since earlier research, reflecting similar experiences of isolation (e.g. Rannard et al., 2005). More effort needs to be made to support these parents through the diagnosis, for example clearer signposting and engaging information on the prognosis of DLD. Parents of children with other neurodevelopmental disorders also experience stress when having difficulty navigating their child’s diagnosis (Bromley et al., 2004; Krakovich et al., 2016; Olsson & Hwang, 2008). As a result, parent-led interventions have been developed (Tarver et al., 2019); parents of children with DLD could benefit from similar support. Simple efforts should also be made to increase visibility of adults with DLD, so that parents and children have a realistic perspective on their futures.

The relative empathetic strengths of children with DLD is suggested in prior literature (Toseeb & St Clair, 2020). What has not been yet documented, however, is their potential creative strengths. Other literature has suggested that the flexible thinking and low inhibitory control that is characteristic of other neurodevelopmental disorders, could contribute to higher levels of creativity (e.g. White & Shah, 2006). This warrants further investigation, particularly as creative initiatives could be used to enable children to express themselves more easily and ultimately thrive.

### Strengths and Limitations

One major strength of the current study is the use of qualitative interviews. As such, parents’ experiences and their treatment priorities were not restricted to specific pre-defined questions. Topics were not limited to assumptions made by the researcher, and new insights could be explored in real-time. Similarly, the use of Patient and Public Involvement (PPI) increases the confidence with which this research can be said to be participant-led, increasing the value of insights (Brett et al., 2014) and supporting collective learning (Staley, 2017). Furthermore, the inclusion of the first-person perspectives of adults with DLD, and their memories of childhood, minimises the likelihood or parental exaggeration, for example due to their own psychosocial symptoms (Krain & Kendall, 2000). Combining parent and first-person perspectives is also important. Parents have been shown to recognise their child’s interpersonal psychosocial difficulties more so than the child themselves (Hebron et al., 2015). As an adult, however, an individual with DLD may be better equipped to reflect on their emotional experience as a child, and the salient moments that may be underestimated by the parent; particularly when the parent is neurotypical.

The study was also successful in engaging families from a range of different backgrounds (regionally, socioeconomically and ethnically), however there are reasons the findings may still exhibit a bias. All participants were mothers, most were white and most were from an above-average income bracket. This lends itself to a societal (being white) and economic privilege in the UK and Ireland that increases their ability to access support (e.g. Durà-Vilà & Hodes, 2012). For example, most families in the sample had been able to manage their finances in order to pay for private help for their child. Additionally, many of the mothers could be referred to as ‘relative experts’. The sample included one clinical psychologist, three teachers and another who was studying developmental psychology, meaning they all had a good understanding of typical child development. Altogether, these characteristics mean the parents are likely to have more knowledge of the public services available and confidence in navigating them than many other parents; those not as societally advantaged may experience even more isolation and stress. Nonetheless, only one parent had heard of DLD prior to their child’s diagnosis; a lack of knowledge that appeared defining of the psychosocial experience of the parents. Importantly, key components and themes were also consistent across the range of economic privilege that this sample did represent, including far less economically privileged adults with DLD (study II).

A further limitation is the lack of formal assessment of DLD amongst the participants, or their individual language needs. As such, the researchers relied on parent-report and self-report alone. There is a recognised heterogeneity amongst the DLD population (Bishop et al., 2016), meaning it would be important to confirm that the individuals within this sample did not just represent a certain subgroup of those with DLD. Nonetheless, it is this heterogeneity that renders uniform assessment difficult and can explain the lack of consensus amongst professionals regarding the best method for assessing DLD (Thomas et al., 2019). It was outside of the temporal and financial capacities of the researchers to conduct adequately thorough and independent language assessments of the participants, something that further research should consider when expanding on these results. Confidence in the results can be ascertained from the similarity between the themes derived from the parent interviews and the reflections from adults who self-reported a range of language needs (mild to very severe).

Similarly, no IQ assessment was conducted of either populations (child or adult). The drawback of assessing IQ as a way of distinguishing individual’s needs is that it is recognised to change over time, and with different measures (Volkers, 2018). Nonetheless, it is important to gather this information in order to compare samples between different studies (Leonard, 2020).

The inclusion of those who are not British-born, and who spent some years of their child’s upbringing in other countries, as well as adults residing in other countries helps increase the generalisability of the results. Nonetheless, the UK and Ireland, where all parents interviewed reside, have similar healthcare systems (Wendt, 2014), including universal free healthcare. Access to these systems could act as a further privilege to many of the sample, meaning those in other countries may experience different challenges in navigating both healthcare and school support for their child.

### Conclusions & Implications

The documentation of anxiety and social frustration amongst children with DLD may not be novel, but many other insights provided by the interviews are. One example is the key contribution of intolerance of uncertainty to behaviours expressing preference for routine and sameness, suggesting the manifestation of anxiety may be similar for children with DLD and those with ASD. Similarly, the combination of negative social experiences and emotion dysregulation appears to result in maladaptive behavioural coping mechanisms, such as avoidance. There is an opportunity for interventions to target these root functions of behavioural problems. As such, interventions can help interrupt the vicious cycle formed between cognition and behaviour. Future research could also look to more specifically draw from the theoretical frameworks used for children with other, more widely understood, neurodevelopmental disorders, such as autism spectrum disorders. Thus, these novel findings for children with DLD can be expanded and built upon, in order to inform intervention development and support.
